# Risk factors for RhD immunisation despite antenatal and postnatal anti-D prophylaxis

**DOI:** 10.1111/j.1471-0528.2009.02244.x

**Published:** 2009-06-17

**Authors:** JM Koelewijn, M de Haas, TGM Vrijkotte, CE van der Schoot, GJ Bonsel

**Affiliations:** a Sanquin Research, Amsterdam, and Landsteiner Laboratory, Academic Medical Centre, University of AmsterdamAmsterdam, the Netherlands; b Division of Public Health, Academic Medical Centre, University of Amsterdamthe Netherlands; c Department of Health Policy and Management, Erasmus Medical CentreRotterdam, the Netherlands

**Keywords:** Prevention, RhD pregnancy immunisation, risk factors

## Abstract

**Objective:**

To identify risk factors for Rhesus D (RhD) immunisation in pregnancy, despite adequate antenatal and postnatal anti-D prophylaxis in the previous pregnancy. To generate evidence for improved primary prevention by extra administration of anti-D Ig in the presence of a risk factor.

**Design:**

Case–control study.

**Setting:**

Nation-wide evaluation of the Dutch antenatal anti-D-prophylaxis programme.

**Population:**

Cases: 42 RhD-immunised parae-1, recognised by first-trimester routine red cell antibody screening in their current pregnancy, who received antenatal and postnatal anti-D Ig prophylaxis (gifts of 1000 iu) in their first pregnancy. Controls: 339 parae-1 without red cell antibodies.

**Methods:**

Data were collected via obstetric care workers and/or personal interviews with women.

**Main outcome measure:**

Significant risk factors for RhD immunisation in multivariate analysis.

**Results:**

Independent risk factors were non-spontaneous delivery (assisted vaginal delivery or caesarean section) (OR 2.23; 95% CI:1.04–4.74), postmaturity (≥42 weeks of completed gestation: OR 3.07; 95% CI:1.02–9.02), pregnancy-related red blood cell transfusion (OR 3.51; 95% CI:0.97–12.7 and age (OR 0.89/year; 95% CI:0.80–0.98). In 43% of cases, none of the categorical risk factors was present.

**Conclusions:**

In at least half of the failures of anti-D Ig prophylaxis, a condition related to increased fetomaternal haemorrhage (FMH) and/or insufficient anti-D Ig levels was observed. Hence, RhD immunisation may be further reduced by strict compliance to guidelines concerning determination of FMH and accordingly adjusted anti-D Ig prophylaxis, or by routine administration of extra anti-D Ig after a non-spontaneous delivery and/or a complicated or prolonged third stage of labour.

## Introduction

Haemolytic disease of the fetus and newborn (HDFN) has long been a major specific cause of perinatal mortality and morbidity.^1^ In 1941, Levine elucidated the process of fetal and neonatal red cell destruction because of maternal red cell antibodies, a process he coined ‘immunisation’.^2^ Rhesus D (RhD) antibodies appeared to be responsible for most cases of severe HDFN.^3^,^4^. In the case of RhD immunisation, the common sequence is a prior pregnancy with an RhD-positive fetus, which induces fetomaternal haemorrhage (FMH)-related immunisation and a subsequent pregnancy with another RhD-positive fetus, which triggers manifest disease.

Administration of anti-D immunoglobulin (Ig) suppresses the immune response of an RhD-negative mother, exposed to RhD-positive fetal cells; the precise mechanism is still unclear.^5^,^6^ Anti-D Ig supposedly induces rapid clearance of RhD-positive fetal cells, interfering with the presentation of the RhD blood group antigen by dendritic cells and macrophages; additionally, it might be that RhD-reactive B cells are suppressed in the production of RhD antibodies.^6^ Routine postnatal administration of anti-D Ig significantly prevents the occurrence of HDFN and has been successfully introduced in developed countries since 1969. Additional guidelines advise administering anti-D Ig in conditions prone to FMH, such as miscarriage, termination of pregnancy, invasive prenatal diagnostic procedures, external version, caesarean section.^7^–^9^ The combined strategy of routine postnatal administration as part of national prevention programmes and of additional anti-D Ig in high-risk conditions during pregnancy and delivery has substantially decreased RhD immunisation in all developed countries. A further decrease was observed in several studies investigating routine *antenatal* administration of anti-D Ig, primarily to prevent immunisation from undetected FMHs during the last trimester of pregnancy.^10^–^12^ But, even if postnatal and antenatal prophylaxis are combined, 0.1–0.3% of women at risk still develop RhD antibodies,^13^,^14^ contributing to a significant number of new RhD immunisations and cases of HDFN. For example, 18 of the 34 new RhD immunisations in parae-1 in 2004 in the Netherlands occurred despite adequate prophylaxis in the previous pregnancy.^14^ If preventable risk factors contributing to remaining immunisation can be identified, a further decrease of HDFN could be achieved. Studies concerning remaining risk factors commonly apply the Kleihauer–Betke test as outcome proxy for immunisation risk; no study has so far identified the risk factors based on actual RhD immunisation in the next pregnancy as primary outcome. Existing studies using the Kleihauer proxy show varying results and provide no evidence for a correlation between large FMH and the incidence of events, accepted as risk factors for FMH, for example, caesarean section.^15^–^19^ Salim *et al.*^19^ performed the largest prospective controlled study on 313 women who underwent caesarean section and 253 women with a vaginal delivery, and did not find any evidence for a relation between the mode of delivery and the rate of large FMH. Differences in the application of the Kleihauer–Betke test (e.g. cut-off point for a positive test, the interval between delivery and the Kleihauer–Betke test) and/or a poor relation between the Kleihauer–Betke test and RhD immunisation risk contribute to this lack of evidence.

The present case–control study aimed to detect risk factors for observed RhD immunisation as detected in the next pregnancy, to optimise prevention programmes for RhD immunisation.

## Materials and methods

### National prevention programme

The Dutch programme for prevention and detection of RhD immunisation in pregnancy is free of charge. All RhD-negative women who deliver an RhD-positive child receive 200 μg (1000 iu) of anti-D Ig within 48 hours of delivery. Antenatal anti-D prophylaxis has been introduced since 1 July 1998. One dose of 200 μg anti-D Ig is administered in the 30th week of pregnancy, which was until May 2008, restricted to RhD-negative women without a living child, because of anti-D Ig scarcity.

An additional dose of anti-D Ig on top of routine anti-D prophylaxis is advised after a miscarriage (>10 weeks of completed gestation), after termination of pregnancy and after invasive procedures or external version during pregnancy. A Kleihauer–Betke test for guiding of doses of anti-D Ig is advised after abdominal trauma during pregnancy and after twin delivery, caesarean section, fundal pressure or surgical removal of the placenta.^8^ All pregnant women are screened for red cell antibodies in the first trimester of pregnancy; RhD-negative women are screened again in the 30th week.^20^ The programme is monitored by an individual data registry (demographic characteristics of the mother, parity, RhD factor, screening results, antenatal and postnatal anti-D Ig administration) at the National Vaccination Offices. The coverage of the prevention programme is close to 100%.^21^

### Study design

We performed a case–control study. Cases were 42 RhD-negative parae-1 with an RhD immunisation, newly detected upon first-trimester screening, who developed RhD antibodies despite the documented administration of adequate antenatal and postnatal prophylaxis during their previous pregnancy and delivery. The cases were identified in a nationwide study, covering 1999, 2002 and 2004, which evaluated the effect of routine antenatal anti-D prophylaxis. The total number of births (from 24 weeks onwards) during these years was 391 000 (National Birth Statistics). The prevalence of new RhD immunisations upon first-trimester screening in parae-1, despite antenatal ánd postnatal anti-D-prophylaxis in the first pregnancy, was 0.31% (39/12 576). The denominator for this prevalence, including all RhD-negative parae-1 who delivered their first child after introduction of antenatal anti-D-prophylaxis in the Netherlands, was calculated from National Birth Statistics (calculation described elsewhere).^14^ During the study period, 37 cases were identified who for sure received antenatal and postnatal anti-D. These cases were included in the current case–control study, as well as five cases identified in 2000 and 2003, as we also collected risk factor data about these cases. Parae-1 who had a negative first-trimester screening test but had a positive screening at or after the 30th week screening, were not included, to restrict our analysis to risk factors during the first pregnancy and delivery only. We included a control group of 339 (RhD-positive and RhD-negative) parae-1 with a negative result of the first-trimester red cell antibody screening, to compare the prevalence of putative risk factors in the case group with the population prevalence. The controls were randomly selected by obstetric care workers between 1 September 2002 and 1 June 2003 in a nationwide evaluation study of the non-RhD Red Blood Cell (RBC) antibody screening programme, described elsewhere.^22^ The controls in this study were matched with the cases (pregnancies with a non-RhD immunisation) for obstetric care worker and gestational age and included around the 20th week of pregnancy. The obstetric workers in primary care (midwives and general practitioners) recruited three controls and obstetricians (clinical care) recruited only one, because of time constraints. From the 968 controls in this evaluation study (the total population of pregnant women during the inclusion period was ±150 000 according to National Birth Statistics), we selected all parae-1 who delivered their first child from 15 September 1998 onwards, at a gestational age of at least 30 weeks (*n* = 339), implicating that the 30th week of pregnancy was after the introduction of the antenatal anti-D prophylaxis programme in the Netherlands. As exposure to potential risk factors is unrelated to the RhD factor of the woman, data from both RhD-positive and RhD-negative women could be used.

All controls gave informed consent for participation in studies concerning pregnancy immunisation. The study was approved by the relevant professional organisations (obstetricians, midwives, general practitioners, paediatricians, clinical laboratories). Representatives of those organisations monitored the study process. Registration data are legally available for scientific research in the Netherlands. Primary study data about the cases were retrieved from existing registries; these data were completed by additional routine care data, obtained from the obstetric care workers. These study procedures do not require individual consent under Dutch law.

### Data collection methods

After primary selection of cases via laboratory registries, additional data were collected about potential risk factors via the obstetric care worker, with a structured questionnaire, mainly in a telephone interview, from July 2004 until April 2005. The source of the risk factor data was the medical record of the previous pregnancy and the record of the current pregnancy, which includes information about the obstetric history. Potential risk factors were related to increased FMH, decreased levels of anti-D Ig or altered immune response (maternal weight, ethnicity and age, paternal ethnicity, gender of the child, twin pregnancy, invasive prenatal diagnostic procedures, external version, postmaturity (≥42 weeks of completed gestation), mode of delivery, surgical removal of the placenta, pregnancy-related RBC transfusion). Similar data were collected from the controls, via the obstetric care worker (40%) or from the pregnant woman in a personal interview by phone by two of the investigators, who were experienced interviewers (60%) (JK, TV). It was not possible to collect valid data about fundal pressure, as this procedure was not always documented in the medical records, especially not in cases with assisted vaginal delivery and women themselves were not always sure whether fundal pressure was given or not. Since ABO blood group incompatibility between mother and fetus can protect against RhD immunisation,^23^ we also evaluated whether there was a difference in ABO blood group distribution between cases and controls. The knowledge about the presence of RhD antibodies theoretically could have induced recall bias. We judge this to be unlikely, as the significant risk factors are well defined.

### Data-analysis

First, univariate analysis of risk factors was performed (Pearson’s chi-square test, Fisher’s exact test (*n* < 5) or Student’s *t*-test, depending on the measurement level of the variable). Next, multivariate logistic regression analysis was performed. Univariate factors shown to be important (*P* < 0.10) were offered stepwise to the model; models were checked for interaction. In case of distorting collinearity (two variables with overlap), we selected the variable most causally connected to the outcome, rather than the one with highest explanatory power. Finally, we estimated the remnant risk for RhD immunisation in the next pregnancy for all possible combinations of significant risk factors by multiplying the Odds ratio, predicted by the model, with the baseline immunisation prevalence of 0.310% in women without significant risk factors and adequate antenatal and postnatal prophylaxis^14^ (supplemental data and calculations available from the authors).

By design, controls under primary care in early pregnancy (with a lower prevalence of potential risk factors such as for example a previous caesarean section) were over-represented, which could contribute to over-estimation of the effect of potential risk factors. We therefore restored the proportion of primary care pregnancies in the control group (298/339 = 88%) to the population proportion for primiparae of 72%^24^ by weighting the primary care controls with 0.35. These weighted data were used in all analyses.

Missing values (<1%) were not substituted. Goodness of fit of the logistic regression models was assessed by the standard Hosmer and Lemeshow test. All statistical analyses were performed in SPSS 11.0 (SPSS Inc., Chicago, IL, USA).

## Results

### Risk factors for RhD immunisation, univariate analysis

As shown in Table 1, postmaturity (≥42 weeks of completed gestation), non-spontaneous delivery (assisted vaginal delivery or caesarean section), pregnancy-related RBC transfusion and the birth of twins, were found to be significant univariate risk factors for RhD immunisation in parae-1 with RhD antibodies detected in first-trimester screening. Caesarean section and assisted vaginal delivery were combined in one variable (non-spontaneous delivery) to increase power. In our view, this is justified because of the same supposed underlying mechanism and the comparable univariate ORs of 2.1 and 1.7 respectively. Unexpectedly, younger age at first delivery was a significant risk factor for RhD immunisation (*P* = 0.02). We have no indications that this was an artefact of our study design, since the mean age of the control group was comparable with the mean age of primiparae in the Dutch population according to published national registry data (29.1 years from 2000 until 2005).^25^ We did not observe a difference in birth interval between the cases (2.57 year) and the control group (2.51 year) either.

**Table 1:** Univariate risk factors for newly detected RhD immunisations in parae-1 with completed anti-D Ig prophylaxis during first pregnancy and delivery

Risk factors in 1st pregnancy/delivery	Cases (*n* = 42)	Controls (*n* = 339*)	*P*-value*
**General**			
Maternal blood group A/AB (%)	40.5	43.2	0.76
Body mass index—mean (SD)	23.8 (4.5)	24.0 (4.5)	0.84
Body weight—mean (SD) in kg	67.6 (11.5)	69.6 (13.3)	0.42
Body weight >75 kg (%)	21.9	23.8	0.82
Body weight >100 kg (%)	3.1	3.3	0.71
Maternal ethnicity non-Dutch (%)	7.3	7.5	0.64
**Previous pregnancy and delivery**			
Maternal age at delivery—mean (SD) in years	27.9 (4.2)	29.4 (3.4)	0.02
Paternal ethnicity non-Dutch (%)	10.8	8.7	0.45
Male child (%)	59.5	48.3	0.21
Twins (%)	4.8	0	0.05
Invasive prenatal diagnostic procedures (%)	2.4	0.7	0.39
External version (%)	0	2.8	0.37
Postmaturity (≥42 weeks of completed gestation) (%)	19.0	5.5	0.01
Non-spontaneous delivery (%)	47.6	29.3	0.03
Caesarean section (%)	23.8	13.0	0.09
Assisted vaginal delivery (%)	23.8	15.8	0.23
Surgical removal of placenta (%)	6.3	4.7	0.50
Pregnancy-related RBC transfusion (%)	14.3	3.4	0.02
**Preceding interval 1st–2nd child**			
Abortion(s) (%)	11.9	13.0	0.85

* Weighted controls: *n* = 146; all *P*-values based on *n* = 146.

A relatively high distribution volume of anti-D Ig prophylaxis, as reflected by high body weight or body mass index, did not emerge as a risk factor.

Before the introduction of anti-D Ig immunoprophylaxis, ABO antagonism between mother and child was observed to decrease the risk for immunisation.^23^ No direct information on ABO-incompatibility between mother and first child was available, but we did not observe an over-representation of mothers with blood group A or AB in the cases; hence, we did not observe any protective effect of maternal blood group O or B. (Table 1). Invasive prenatal diagnostic procedures and external version in a previous pregnancy, as well as a miscarriage or termination of pregnancy after the first completed pregnancy and delivery—all risk factors for FMH where guidelines prescribe additional anti-D Ig administration—did not emerge as risk factor (Table 1). All but one abortions were spontaneous miscarriages; one termination of pregnancy was observed in the case group of which the duration of pregnancy and administration of anti-D-prophylaxis were unknown.

Non-Dutch ethnicity of the mother or father were not risk factors for RhD immunisation in parae-1. It has previously been reported that male children are more frequently affected by RhD immunisation,^26^ but in our study, the sex of the first child did not significantly influence the immunisation risk although a slightly higher incidence of a prior male child was observed in the cases (Table 1).

### Risk factors for RhD immunisation, multivariate analysis

From the multivariate analyses, postmaturity (OR 3.07). caesarean section or assisted vaginal delivery (OR 2.23) and age at first delivery (0.89/year) emerged as significant risk factors for RhD immunisation; pregnancy-related RBC transfusion was almost significant (OR 3.51); see Table 2. The risk factor ‘twins’ was, despite the *P*-value of 0.05 in the univariate analysis, not offered to the multivariate models, because the prevalence in the control group was 0%. We could not establish any interaction between risk factors.

**Table 2:** Multivariate risk factors for newly detected RhD immunisations in parae-1 with completed anti-D Ig prophylaxis during first pregnancy and delivery

	OR	95% CI
Postmaturity (≥42 weeks of completed gestation)	3.07	1.02–9.20
Caesarean section or assisted vaginal delivery	2.23	1.04–4.74
Maternal age at delivery (years)	0.89	0.80–0.98
Pregnancy-related RBC transfusion	3.51	0.97–12.7

*R*^2^ = 0.150.

In 43% of the cases, none of the significant categorical multivariate risk factors such as non-spontaneous delivery (caesarean section or vaginal assisted delivery), postmaturity and RBC transfusion were present (‘spontaneous’ cases), compared to in 65% of the controls.

To calculate the risk for RhD immunisation in the next pregnancy of combinations of risk factors, we dichotomised age at previous delivery in two groups: younger and older than the mean age in the control group (29.35 years). The calculated immunisation risk (point estimate) varied from 0.2% (95% CI: 0.005–0.33%) in women without risk factors to 2.0% (95% CI: 1.57–3.46%) if all significant risk factors are present (Table 3).

**Table 3:** Model-based risk for RhD immunisation in next pregnancy with completed anti-D Ig prophylaxis in the previous pregnancy, according to the presence of risk factors

	Age at previous delivery > 29.3 years		Age at previous delivery < 29.3 years	
	Duration of pregnancy <42 weeks	Duration of pregnancy ≥42 weeks	Duration of pregnancy <42 weeks	Duration of pregnancy ≥42 weeks
**Spontaneous delivery**				
No RBC transfusion	0.2%	0.6%	0.4%	1.0%
RBC transfusion	0.7%	1.3%	1.0%	1.6%
**Assisted or surgical delivery**				
No RBC transfusion	0.4%	1.0%	0.7%	1.3%
RBC transfusion	1.1%	1.7%	1.4%	2.0%

## Discussion

Our study is the first providing direct evidence about causative risk factors for verified RhD immunisation in the next pregnancy, despite adequate antenatal and postnatal anti-D prophylaxis, given as single doses of 1000 iu (200 μg). A relatively young age at first delivery, non-spontaneous delivery (caesarean section or assisted vaginal delivery) and postmaturity of the previous pregnancy emerged as *independent* risk factors that significantly contributed to the development of RhD antibodies. Pregnancy-related RBC transfusion, despite its lower confidence limit of 0.97, can most likely be added to this risk set. The expected risk factor of twins was established in the univariate analysis, but could not be analysed in the multivariate analysis because twins were missing in our control group.

A non-spontaneous delivery (caesarean section or assisted vaginal delivery) can be considered as a risk factor for increased FMH, exceeding the amount of fetal cells that can be neutralised by a single gift of anti-D Ig of 1000 iu (200 μg). Most national guidelines advise to quantify the FMH by a Kleihauer–Betke test after caesarean section to guide the dosage of postnatal anti-D Ig.^7^–^9^ However, until now there was no clear evidence for this policy. Several cohort studies showed no correlation between assisted delivery and the presence of FMH, but none of these studies provided data about subsequent immunisation^15^–^19^,^27^ Our results suggest that, at least in the above mentioned studies, the sensitivity and the specificity of the Kleihauer–Betke test were apparently insufficient in demonstrating increased FMH after a non-spontaneous delivery. This can be explained in several ways. First, the application of the Kleihauer–Betke test (e.g. the selected cut-off point for a positive test) varied between the studies. Second, to detect risk factors for a relatively rare condition as a (large) FMH, a cohort study is not the most appropriate study design, because large numbers of women with a potential risk factor have to be included to achieve enough power. Third, one could argue that the Kleihauer–Betke test misses some FMHs relevant for immunisation. The Kleihauer–Betke test can be performed manually with stained blood smears or using a flow cytometry-based method and the technical merit and validation of the used method may be of influence on test performance.^28^ Furthermore, fetal blood loss into the maternal intraperitoneal cavity, hence not detectable by the Kleihauer–Betke test, might well lead to RhD immunisation.

The postmaturity risk factor might be explained by insufficient levels of preventive anti-D Ig, after a single gift of 1000 iu (200 μg) in week 30 of pregnancy, in addition to the obvious immunogenic effect of prolonged exposure to fetal cells. Several studies showed undetectable levels of anti-D Ig more than 12 weeks after administration, even when 300 μg of anti-D Ig was administered in a single gift;^29^ therefore, anti-D Ig levels may drop too low if a pregnancy exceeds 42 weeks.^30^–^32^

RBC transfusion is a less obvious factor contributing to RhD immunisation. Most likely, the RhD-negative RBCs have been given in all cases, as false-negative typing of donors is extremely rare.^33^ The most likely explanation in our view rests on spurious association: RBC transfusions are usually given to women after complicated delivery or prolonged labour, especially a prolonged third stage, with concomitant higher FMH risk. Alternatively, we can think of transfusion-induced triggering of the immune system, in general, stimulating the response to the fetal cells, which is supported by the observation that two out of six transfused women also had developed non-Rh antibodies [anti-K, anti-Fy(a)], compared with none of the RhD immunised women without a RBC transfusion (*P* = 0.017).

Finally, the independent effect of age at time of delivery on the RhD immunisation risk is difficult to explain. It is known that elderly people have diminished immune responses to vaccines and to solid organ transplantations,^34^,^35^ but an effect of age in this healthy young group of women leading to an increased immunisation risk, is poorly understood. Theoretically, the effect could be caused by indirect factors, not covered by our study, that are related to maternal age and immunisation risk.

Our study on risk factors for manifest RhD immunisation, instead of on factors related to the immunisation pathway (FMH), identified preventable risk factors and in our view provides opportunities to decrease the incidence of RhD immunisation. Assisted vaginal delivery and pregnancy-related RBC transfusion should be added to the set of risk factors for RhD immunisation in the current guidelines. In the presence of a risk factor, two policies are to be considered: administration of a standard extra dosage of anti-D Ig or testing for FMH, followed by adjusted anti-D Ig prophylaxis. Considering the first policy, it is reassuring that clinical conditions where standard *additional* anti-D Ig is universally prescribed, such as spontaneous miscarriage, termination of pregnancy, invasive procedures during pregnancy and external version, did not emerge as a risk factor in our analysis. However, it should be kept in mind that our sample size was too small to establish a difference in infrequent risk factors such as invasive diagnostic procedures. Whether the second policy will decrease the RhD immunisation rate cannot be concluded from our study. In none of the cases of assisted or surgical delivery in our study, a Kleihauer–Betke test was performed and it seems plausible that some cases might have been prevented if this test had been performed, followed by adjusted anti-D Ig administration. However, we have no data about the application of the Kleihauer–Betke test and its relation to immunisation status later. Another consideration is the practicability of such a test-based approach of additional anti-D Ig administration. The lack of testing we observed in our cases, even after a caesarean section, is in contrast with the current Dutch guidelines, which suggest (rather than prescribe) a Kleihauer–Betke test.^8^ Replies to an e-mail questionnaire, without reference to our observations, under half of all obstetric partnerships in the Netherlands (response rate 70%), showed that about 50% of them do not perform a Kleihauer–Betke test and administer the standard dosage anti-D Ig after a caesarean section or after fundal pressure; after an assisted vaginal delivery, >90% of the obstetricians do not test and provide the standard dose of anti-D Ig (J.M. Koelewijn, unpubl obs.). In view of this observation and of the fact that not all hospital laboratories are experienced in the performance and interpretation of the Kleihauer–Betke test, routinely administration of extra anti-D Ig in the presence of a risk factor might be considered.

Another option to minimise the risk for RhD immunisation may be proposed. Splitting of the single dose of 200 μg or 300 μg (1000 or 1500 iu respectively) of anti-D Ig into two gifts, in week 28 and in week 34 respectively, will theoretically have a cumulative effect on anti-D Ig plasma levels, which might contribute to sufficient levels of anti-D Ig in postmature pregnancies, hence to a decreased immunisation risk. As far as we know, this theoretical consideration is not supported by research data. Furthermore, there is evidence that compliance with a two-dose regimen is less than ideal.^36^,^36^,^37^ Thus, splitting of the dosage can in our view not be recommended at this moment. Another option would be an additional dose of anti-D Ig in week 40 or 41, but there are no data to support the efficacy of such a practice. The aforementioned measures will be important in reducing the remaining incidence of RhD immunisation, especially in a prevention programme with a relatively small dose of anti-D Ig and in countries without routine antenatal prophylaxis,. This would in our study have been applicable in 57% of current failures. If all immunisations in the next pregnancies after a non-spontaneous first delivery and or pregnancy-related RBC transfusion (one-third of all first deliveries) could be prevented by administration of a standard extra dose of anti-D Ig or by test-guided administration of extra anti-D Ig, the Number Needed to Treat would be ±110, compared to ±20 for routine postnatal anti-D prophylaxis and ±350 for routine antenatal anti-D prophylaxis.^14^ However, 43% of the failures cannot be explained by risk factors associated with an increased FMH and/or too low levels of anti-D Ig. From the biological point of view, variations in individual immune response and the influence of age are intriguing topics, needing further research.

In conclusion, our risk factor analysis provides indisputable targets to reduce RhD immunisations and subsequent HDFN at an apparently low practical and financial price, at least in the Netherlands.
